# Spectral reflectance of marine macroplastics in the VNIR and SWIR measured in a controlled environment

**DOI:** 10.1038/s41598-021-84867-6

**Published:** 2021-03-08

**Authors:** Mehrdad Moshtaghi, Els Knaeps, Sindy Sterckx, Shungudzemwoyo Garaba, Dieter Meire

**Affiliations:** 1 VITO Remote Sensing, Mol, 2400 Belgium; 2 Marine Sensor Systems Group, Institute for Chemistry and Biology of the Marine Environment, Carl von Ossietzky University of Oldenburg, Wilhelmshaven, 26382 Germany; 3 Flanders Hydraulics, Antwerp, 2140 Belgium

**Keywords:** Environmental sciences, Ocean sciences, Optics and photonics

## Abstract

While at least 8 million tons of plastic litter are ending up in our oceans every year and research on marine litter detection is increasing, the spectral properties of wet as well as submerged plastics in natural marine environments are still largely unknown. Scientific evidence-based knowledge about these spectral characteristics has relevance especially to the research and development of future remote sensing technologies for plastic litter detection. In an effort to bridge this gap, we present one of the first studies about the hyperspectral reflectances of virgin and naturally weathered plastics submerged in water at varying suspended sediment concentrations and depth. We also conducted further analyses on the different polymer types such as Polyethylene terephthalate (PET), Polypropylene (PP), Polyester (PEST) and Low-density polyethylene (PE-LD) to better understand the effect of water absorption on their spectral reflectance. Results show the importance of using spectral wavebands in both the visible and shortwave infrared (SWIR) spectrum for litter detection, especially when plastics are wet or slightly submerged which is often the case in natural aquatic environments. Finally, we demonstrate in an example how to use the open access data set driven from this research as a reference for the development of marine litter detection algorithms.

## Introduction

Plastic material offers numerous properties such as lightweight, resilience, resistance to corrosion, color, and ease of processing, which makes them attractive for many applications^1–3^. Manufacturing of plastic materials at low costs has been found to be a major source of the plastic waste as most of the consumer goods are packaged in single use plastics. However, these plastics are not biodegradable, and their durability and strength makes them a serious environmental contaminant^4,5^. A large amount of this plastic waste finally ends up in the aquatic environment. It is estimated that more than 150 million tons of plastics have accumulated in the world’s oceans, while 4.6–12.7 million tons^6^ are added every year; costing over 2 trillion US dollars^7^. Given the size of the aquatic environment, there is little scientific evidence-based information available on the distribution, types and sources of this marine plastic debris. Marine plastics are mainly monitored by visual surveys from ships, using plankton Neuston net trawls, as well as ingested counts found in marine biota^8^. For example, the JRC exploratory project RIMMEL acquired information about litter, mainly plastic waste, entering the European Seas through river systems using data collected by visual observations over a period of 1 year^9^. The visual survey revealed plastic specimens identified were mostly single use bottles and carry bags, 7 of these materials observed were among the top ten items found in global litter.

Optical sensors on satellites, aircrafts, unmanned aerial systems, drones and handheld devices can contribute to the monitoring of slightly submerged and floating marine plastics^10–12^. These remote sensing technologies have capabilities to generate standardized objective repeated plastic relevant measurements over large geo-spatial areas at sustainable operational costs. It is however limited to the monitoring of aggregated marine plastics, and should be seen as complementary to ship-borne net trawl and visual surveys. Already, several airborne surveys looking for marine plastics have been realized with mainly visual interpretation of the true color RGB or SWIR hyperspectral imagery^13–16^. Over the Great Pacific Garbage patch, very high geo-spatial resolution RGB and SWIR imagery was collected at the same time as trained human observers manually counted visible litter typically diameter above 0.5 m^13,15^. There are challenges with visual detection that include observer bias^17^, misidentification of particles similar to organic matter, or under detection of particles that are too small to be detected by the human eye^18^. Ideally, automated detection approaches could be explored as they might have the potential to improve monitoring of the marine plastic litter.

Knowledge about diagnostic spectral features of virgin or weathered plastics is vital as the demand is increasing for remote sensing technologies relevant to monitoring of marine litter. The waste management and recycling industry has been at the forefront of sensor development, they have robust tailor-made sensors that automatically classify plastic by polymer type, colour and even shapes^19–22^. The chemical composition of the plastic polymers is derived from the spectral measurements in the SWIR (1000–2500 nm) to MWIR (2500–5000 nm) spectrum^23–25^ as well as LWIR (6000–14,500 nm)^26^. However, identifying plastic materials in the ocean, in waterways and harbors is much more challenging than in a controlled industrial environment (e.g. on a conveyor belt). The plastics can be floating on the surface, but can be wet and can be partly or fully submerged. Furthermore, there are features at the sea surface such as whitecaps, sea foam and bubbles generated by wind and waves which can mask the spectral signature from the plastics^27,28^. The water is absorbing strongly the light in the NIR and SWIR and other water constituents such as suspended particulate matter alter the reflected signal^29,30^. Moreover, there is also a contribution from the atmosphere which also alters the signal received at the sensor. Finally, water and small individual plastics can be in same pixel complicating the detection. Garaba and Dierssen^31^ performed some experiments on harvested plastic samples taken to the laboratory (items such as buoys, bottle caps, containers, ropes and nets) and on virgin pellets (PVC, PA 6.6 and PA 6, LDPE, PET, PP, PS, FEP, ABS, Merlon, PMMA). The study revealed the presence of diagnostic absorption features in the NIR-SWIR spectrum centred around 931 nm, 1045 nm, 1215 nm, 1417 nm, 1537 nm, 1732 nm, 2046 nm and 2313 nm. They reported a decrease in reflectance magnitude after the dry plastics were dampened.

In this paper, we further contribute to the scientific evidence-based about marine plastic litter by analyzing the spectral reflectance in the 350 to 2500 nm spectral range of samples measured in a controlled environment simulating clear to turbid waters. The experimental setup in this study was suitable for investigating spectral reflectance changes as a function of the water depth and concentration of suspended material. Furthermore, appropriate or diagnostic spectral wavebands relevant for the detection and distinguishing plastics from other optically active material in coastal and estuarine environments were evaluated.

## Results

In this section, we analyze the spectral reflectance from the experiments in the VITO calibration facility and a water tank; We also demonstrate how this spectral database of plastics can be used to evaluate marine plastic detection algorithms. Here we present an example of a Sentinel-2 based detection algorithm.

### Dry virgin and marine-harvested plastic samples

**Figure 1:**
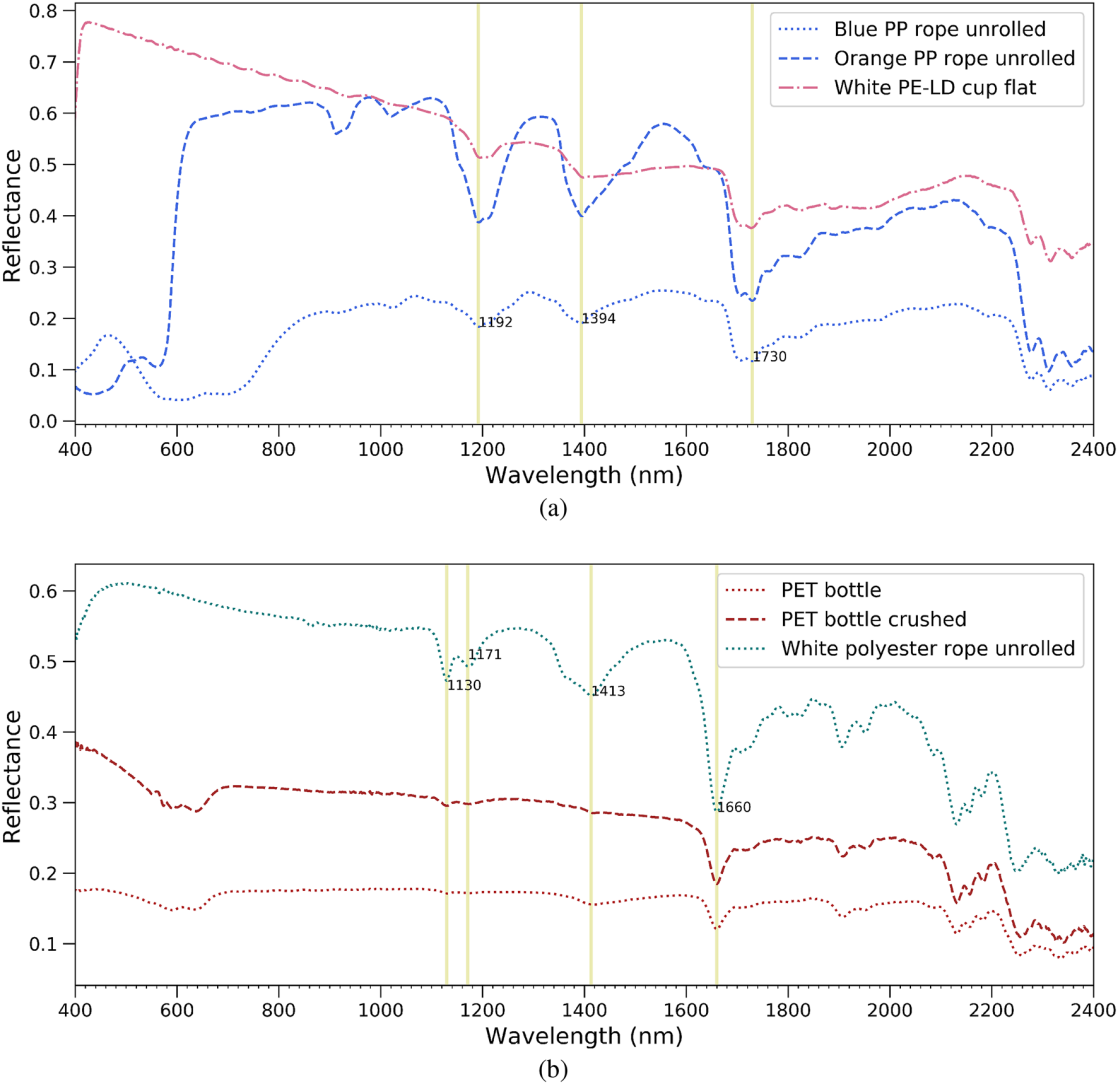
Spectral reflectance of dry virgin (**a**) unrolled Polypropylene (PP) ropes, a Low-density polyethylene (PE-LD) cup (**b**) Polyethylene terephthalate (PET) water bottles and a Polyester (PEST) rope. Absorption features are highlighted by the vertical yellow lines.

The virgin plastics included several samples with known polymeric composition: crushed polyethylene terephthalate (PET) bottles, blue and orange polypropylene (PP) rope, white polyester rope and low-density polyethylene (PE-LD) cup. Their spectral reflectance is shown in (Fig. 1) and the main absorption features in (Table 1). The spectral shape of reflectances in the visible spectrum was consistent with the apparent colour of the plastics. Although having the same polymer composition, the blue and orange rope had different spectral shapes in the visible. To retrieve information about the polymer composition, we have to inspect the SWIR spectral region (1000 nm to 2500 nm). Despite the fact that absolute SWIR reflectance differs, both orange and blue PP ropes show clear absorption features at 1192, 1394 and 1730 in Fig. 1a. PET bottles both crushed and non-crushed ones have a strong feature at 1660 nm and a small feature at 1130 nm. Features around 1730 nm and 1660 nm have been reported before^20^ and are often used to discriminate between different polymers.

**Table 1:** Main absorption features of different polymers.

Polymer	Type of plastic	Main spectral absorption features
Polyethylene terephthalate (PET)	Water bottle	1130 and 1660 nm
Polypropylene (PP)	Rope	1192, 1394, 1730 nm
Polyester (PEST)	Rope	1130, 1413, 1660 nm
Low-density polyethylene (PE-LD)	Cup	1192, 1394, 1730 nm

**Figure 2:**
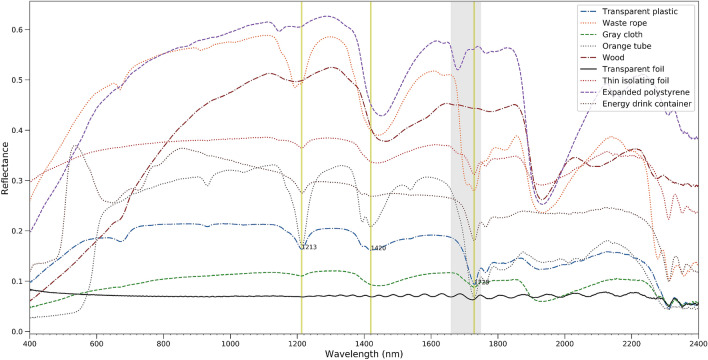
Spectral reflectance of dry natural and anthropogenic materials harvested in the Port of Antwerp in 2019.

Litter obtained from the Port of Antwerp had variable spectral shapes and magnitude (Fig. 2). The measured reflectance was as high as 0.6 in the SWIR (e.g. Expanded polystyrene) and as low as 0.1 (transparent foil and grey cloth). Several samples have a similar shape in the VIS part of the spectral because of their greyish color. In the SWIR, several samples show prominent absorption features (e.g. around 1729 nm, 1213 nm and 1420 nm), others, such as the grey cloth and the transparent foil, have a much flatter reflectance spectrum. The transparent foil has extremely low reflectance and exhibits a sinusoidal pattern due to thin film interference. Hence, discrimination of different plastics based on their absorption features might be complex as it is also likely dependent on the thickness of the plastic material and degree of weathering. Goddijn-Murphy et al.^32^ also showed decreases in reflectance and depths of the absorption bands with increasing transparency of plastic. They considered different buoyant plastics including (1) white, opaque EPS building foam, (2) white semi-transparent HDPE milk bottles, and (3) clear transparent PET soft drink bottles.

### Wet plastics

**Figure 3:**
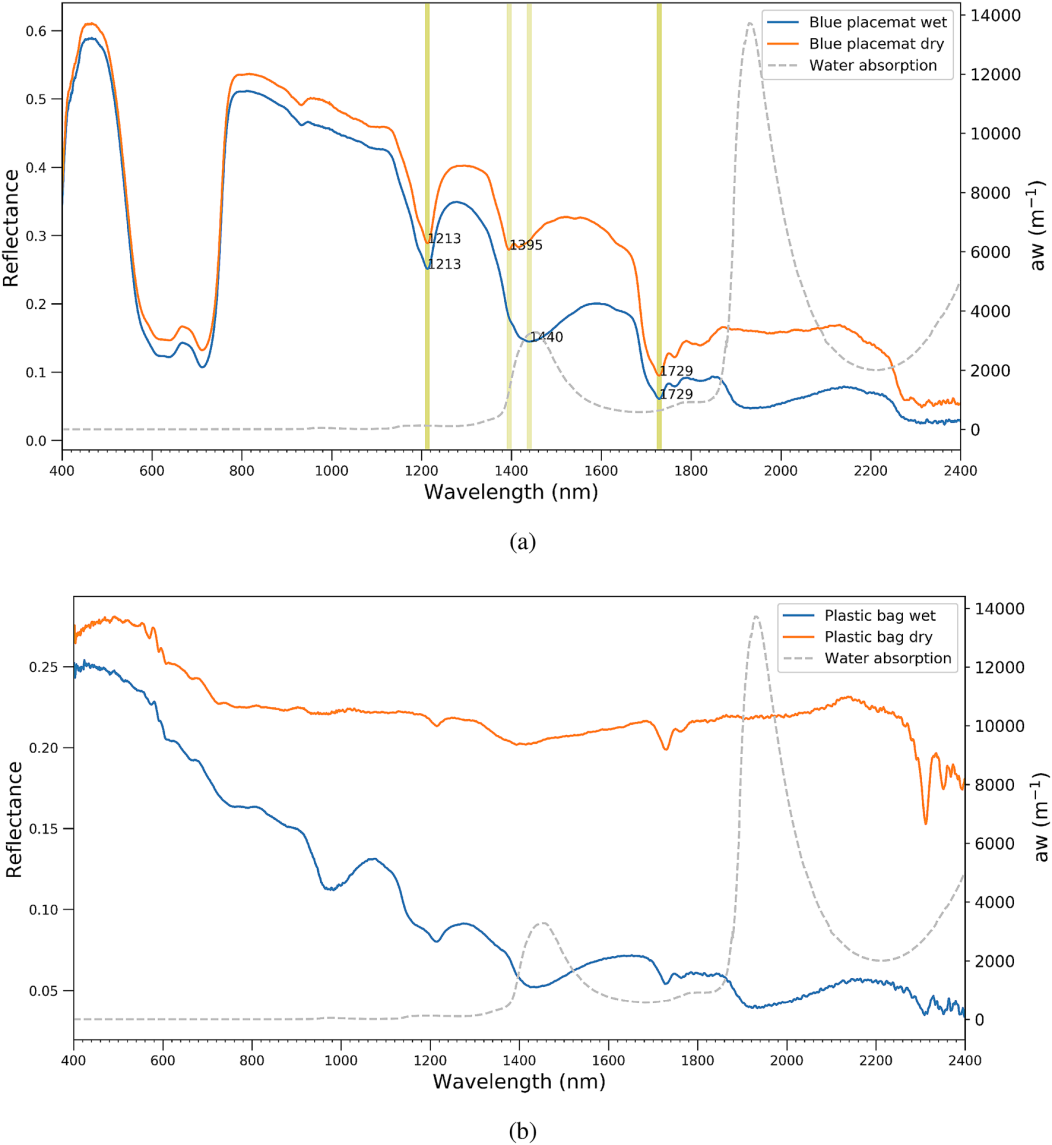
Absorption coefficient of pure water^30,33^ and spectral reflectance of (**a**) dry and wet placemats (**b**) dry and wet plastic bag.

Dry virgin samples have been wetted to analyze the effect of wetness on the spectral responses. Figure 3a illustrates spectral measurements for the wet and dry blue placemat; the pure water absorption coefficient^30,33^ is added as a reference. In the visible wavelength range, wetness does not have pronounced effects on the spectral reflectance because pure water absorption is still relatively low. For the plastic bag in Fig. 3b, differences in the visible range can be explained by the non-uniform surface of the bag, measuring slightly different areas of the bag in wet and dry conditions. In the NIR and SWIR, the effect is much more apparent and reflectance is clearly lower when plastics are wettened. The percentage reduction follows the shape of the pure water absorption coefficient. The effect of wettening is stronger at longer wavelengths and prominent peaks in the pure water absorption also influence the wet plastic reflectance signal. Table 2 shows the reduction percentage from dry to wet for different plastic samples.

Garaba and Dierssen^11^ reported a reduction percentage on average by $56\pm 23\%$; they found it increases with wavelength from 12% in the UV to almost 90% in the SWIR. We see the same pattern in Table 2, but at the same time the signal reduction is very different per sample; for instance, at 2200 nm, it’s 75.33% for orange placemat while it is 16.91% for an orange rope. The capacity to absorb moisture from the environment (moisture absorption) is different per polymer, which is one of the reasons for observing various reduction percentages^34^. Moreover, non-uniformity of plastic targets combined with possible different footprints between dry and wet measurement might explain variation observed between the same type of material.

Wettening also changes slightly the shape of the plastic reflectance spectrum, particular in areas with strong increases or decreases in the pure water absorption coefficient. For a blue placemat in Fig. 3a, the feature at 1395 nm in dry condition shifts to 1440 nm in a wet condition due to the water absorption. Two other features around 1213 nm and 1727 nm are still the same in the wet and dry condition because the first derivative of the water absorption spectrum is near to zero at these wavelengths. Moreover, decrease of reflectance in wet condition comparing to dry is different for samples and can be sharp like the plastic bag in Fig. 3b.

**Table 2:** Relative percentage loss in spectral reflectance, from dry to wet, placemats and ropes.

Wavelength (nm)	Orange placemat	Blue placemat	Blue rope	White rope	Orange rope
931	18.41	6.09	2.98	5.34	− 3.87
1215	33.65	13.01	10.85	12.37	5.23
1732	63.08	35.45	12.65	6.39	9.02
2200	75.33	51.05	23.17	12.83	16.91

**Figure 4:**
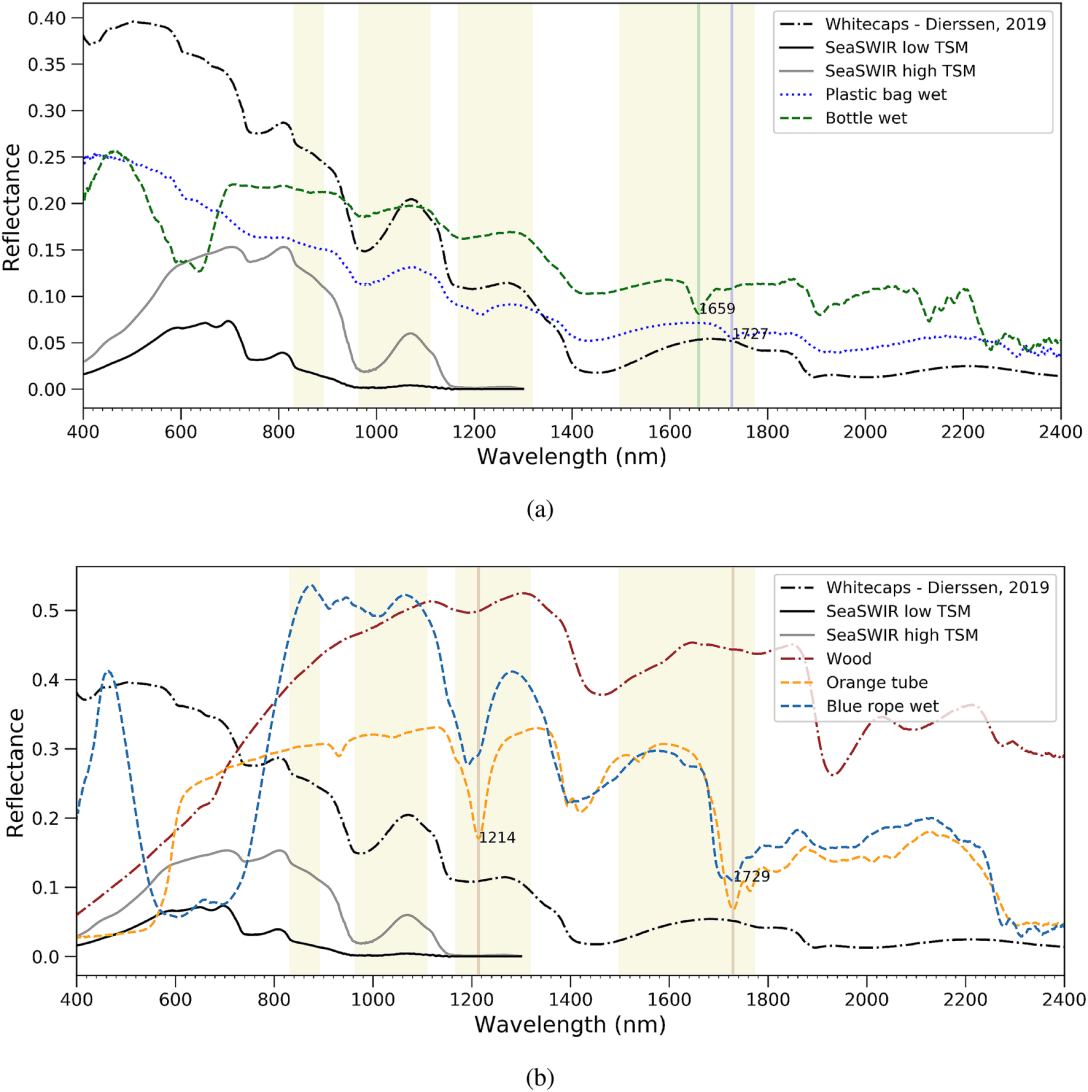
Spectral reflectance of natural and anthropogenic materials (**a**) whitecaps modelled after^27^, SeaSWIR measurements of Total Suspended Matter^35^, wet plastic bag and water bottle (**b**) wood, orange tube and wet blue rope. The vertical line indicate major absorption features.

To evaluate how the presence of suspended sediments in the water and the presence of whitecaps complicate the identification of marine plastic litter, measured and modelled spectra were used. Turbid water reflectance spectra from the SeaSWIR database^35^ and a whitecap spectrum^27^ in Fig. 4 was added next to wet plastics and wood spectra. In the VIS wavelength range, several plastics can be discriminated based on their color from the water. This is the case for e.g. the white transparent plastic bags and plastic bottles (Fig. 4a), which are often found in these waters. Moreover, as can be seen in Fig.  4b, orange-brownish plastics, will be difficult to discriminate from the turbid water in VIS, while a blue rope can be distinguished easily at this range. Although transparent plastic bags can be differentiated from the water itself, they seem almost impossible to discriminate from whitecaps.This means we cannot only look at the spectral information but need information on the geometric shape to distinguish.

Around 1070 nm, all plastics have a significantly higher reflectance than the water, and color is not important anymore. However, we will show later on that this is not true when the plastics are submerged. The reflectance of whitecaps and water was close to zero beyond 1400 nm. We have already seen before that solid plastics like a tube have a high SWIR reflectance which offers an opportunity for discrimination of these types of plastics at this wavelength range. Wood, with its brownish color is difficult to discriminate from the water and the brownish plastics in the VIS, but will be easy to distinguish from bags and bottles in the SWIR. Discriminating wood from solid plastics in the SWIR might be possible based on the absorption features at 1729 nm where wood does not have any feature (Fig. 4b). Finally, when selecting appropriate wavelengths for plastic identification from a remote sensor, the atmospheric transmittance should be taken into account which is highlighted by yellow where transmission is high. Clearly, wavelengths in the VIS, around 1070 nm, NIR around 1214 nm and in the SWIR around 1659 and 1729 nm are suitable.

### Submerged plastics

**Figure 5:**
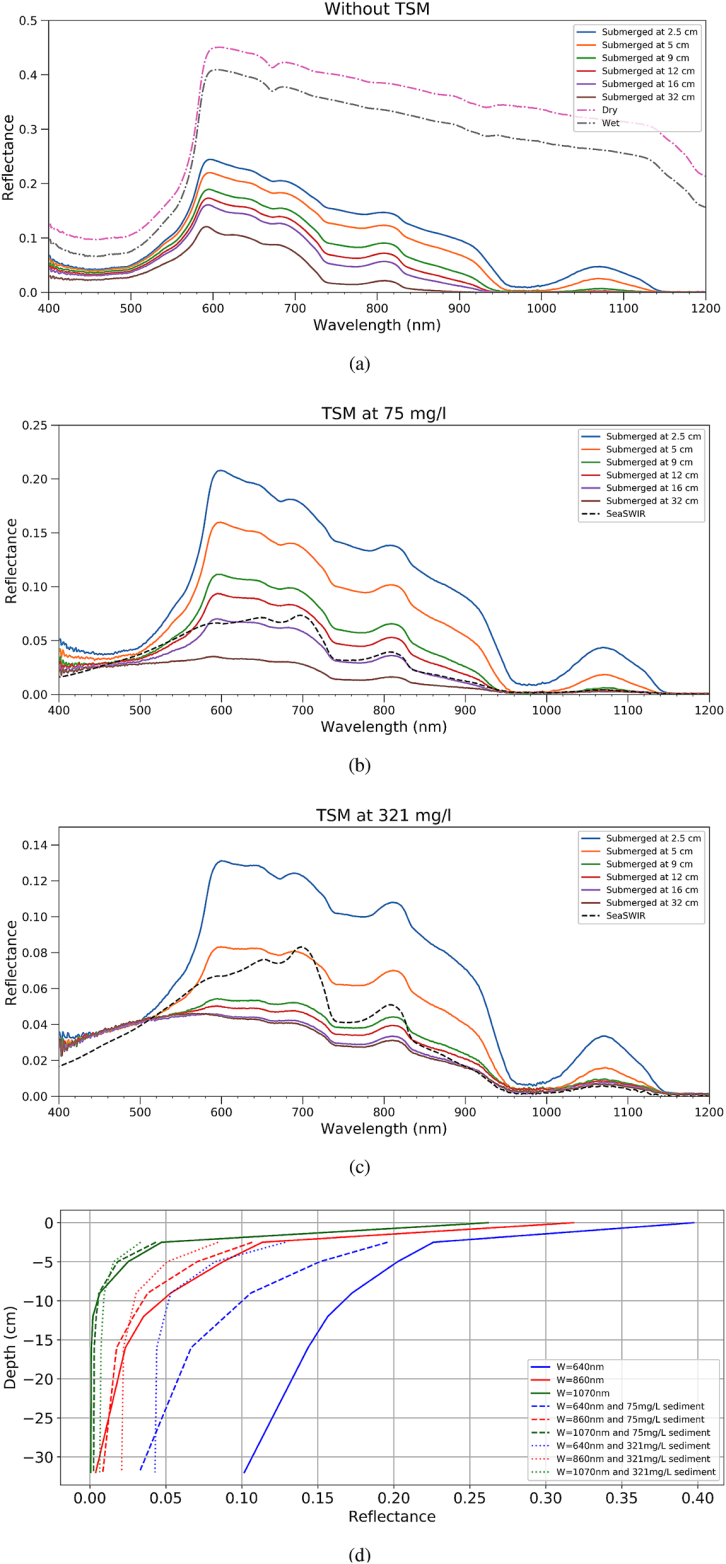
Above water reflectance when submerging an orange rope at different depths in the tank in three conditions: (**a**) without sediment, (**b**) TSM concentration of 75 mg/l and (**c**) TSM concentration of 321 mg/l. A SeaSWIR water spectrum with similar TSM concentration is added. (**d**) Above water reflectance at specific wavelengths for an orange placemat at different depths.

Figure 5 shows spectral reflectance of the orange rope when submerged in clear water at different depths and various TSM concentration. The selected SeaSWIR^35^ spectra have a TSM concentration similar to the TSM concentration in the tank. For instance, in the Fig. 5b a SeaSWIR spectrum with a TSM concentration of 74.44 mg/l was used, while for Fig. 5c a spectrum with a TSM concentration of 300.69 mg/l was used, which were the closest one to TSM concentration in the tank. In Fig. 5a it can be seen that with the slightest submersion of 2 cm all reflectance beyond 1100 nm is absorbed by the water, confirming plastics are almost only detectable at VNIR at this depth. The peak at 1070 nm disappears first when plastics are submerged more than 5 cm. As depth increases, the NIR signature weakens as well, which is even more pronounced severe with more sediment in the tank. Looking at Fig. 5c, the orange rope cannot be discriminated from the turbid water (with a TSM concentration of 321 mg/l) when submerged more than 5 cm. At a TSM concentration of 75 mg/l, the water itself has a similar reflectance than an orange rope submerged at 16 cm (Fig. 5b).

Finally, Fig. 5d presents the spectra of the orange placemat submerged in water with a TSM concentration of 75 and 321 mg/l at 640,860 and 1070 nm. These wavelengths were chosen because they correspond with very different values of the pure water absorption coefficient (respectively 0.3, 4.5 and 14.1 m^-1^). Clearly reflectance decreases fastest with depth at 1070 nm because of the highest pure water absorption coefficient. The effect of the TSM concentration is however much less at 1070 nm. The effect of the TSM concentration is largest for the red wavelength at 640 nm.

### Evaluation of plastic detection indices

**Figure 6:**
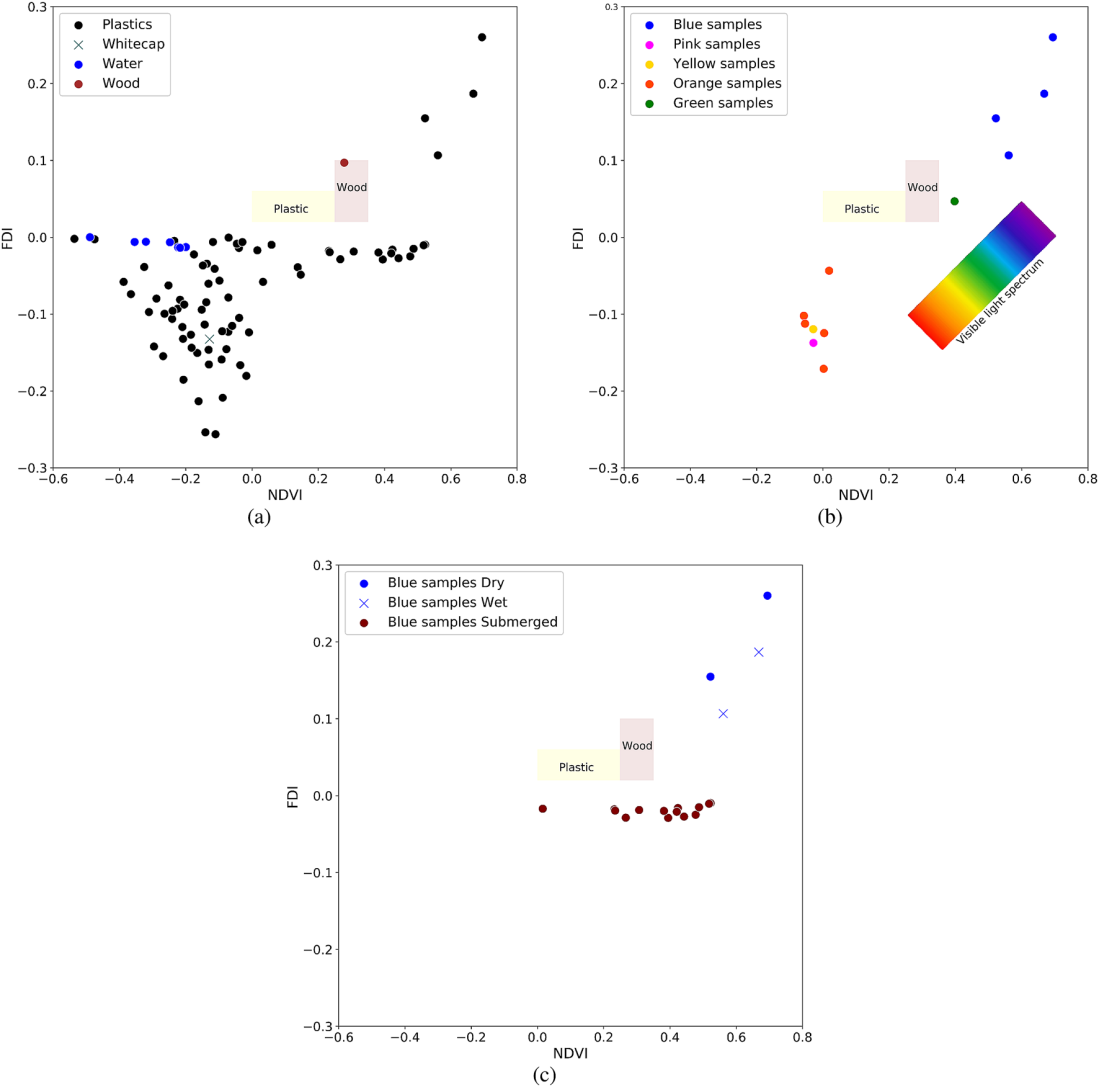
FDI and NDVI for (**a**) plastic samples, wood, whitecap and water, (**b**) different colors and (**c**) effect of submersion. Yellow and brown rectangular correspond to Biermann’s proposed range for plastic and wood respectively. Water and all submerged data are collected in zero, 75 and 321 mg/l sediment condition.

The dataset of dry, wet and submerged spectral measurements can be used to test plastic detection algorithms. Here we test an algorithm for finding plastic patches from Biermann et al.^36^ which is based on the Normalized Difference Vegetation Index (NDVI) and Floating Debris Index (FDI). Figure 6 shows scatterplots of the NDVI versus the FDI for different plastic types in wet, dry and submerged conditions. Reported ranges for plastic and wood from Biermann et al.^36^ are added as a reference in yellow and orange boxes. In Fig. 6a indices for the wood sample are within Biermann’s timber range for NDVI and FDI. The plastic samples, however, show much more diversity compared to their results. The water spectra in Fig. 6b have different TSM concentration which is the reason for having several points for water. When sediment concentration is increasing, NDVI of the water spectrum is increasing as well and it is shifting to right in the graph. As can be seen in Fig. 6c, even by slightly submerging of a plastic sample, the FDI decreases significantly and goes towards zero which makes that the indices are different than proposed in Bierman et al.^36^.

**Figure 7:**
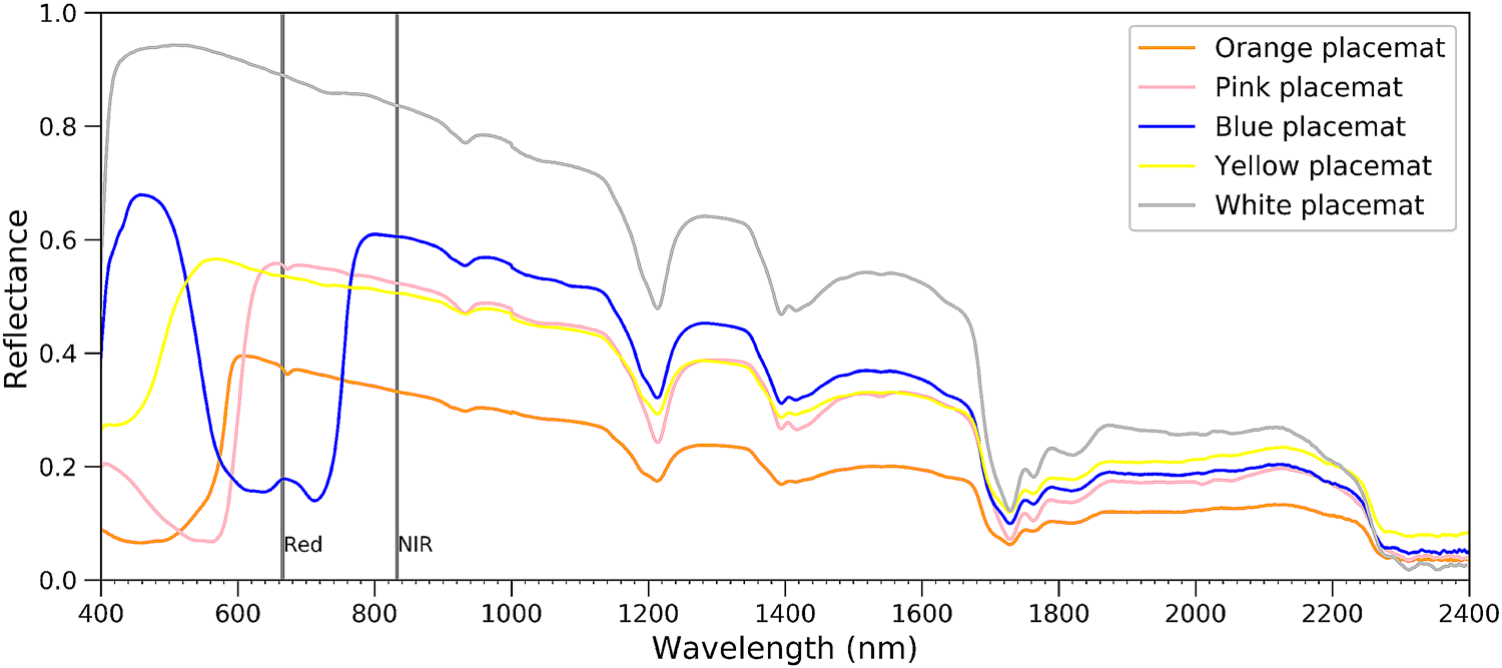
Spectral reflectance of dry virgin placemats in different colors.

Highest NDVI and FDI can be seen for blue plastics in both dry and wet conditions. Considering other colors in Fig. 6b, the same pattern as the visible light spectrum can be seen where red and yellow samples have low NDVI and FDI, while green samples have a higher NDVI and FDI. This is due to the NDVI formula, as it just uses the NIR and red band ; therefore it can be affected by the color of samples. Figure 7 shows the spectrum of the placemats with different colors with same size and shape in dry condition highlighting red and NIR wavelengths.

The relationship between the NIR and Red band has a direct impact on the NDVI. As can be seen from Fig. 7, the blue placemat has minimum reflection amongst the different colors while it has high NIR reflectance. This is the reason why we see highest NDVI for blue and green samples in Fig. 6b. Although FDI uses different bands, the same explanation holds, where blue samples have the highest FDI. These results show, that plastic reflectance is much more diverse than presented in Biermann et al.^36^. Different types of plastics have different values for the indices, but also immersion and wettening has a profound effect. For their specific cases, the proposed NDVI/FDI index might work, but it seems the performance highly depends on the types of plastics, and concentration of suspended sediments.

## Discussion

The spectral reflectance (350–2500 nm) of plastic samples was studied in wet and dry condition, with the samples submerged in water at different depths and with different TSM concentrations. Whitecaps and wood, often found floating on the water surface and mixed with the plastics, have been considered as well. This information is essential for designing a new sensor or selecting an existing one to detect plastics and discriminate plastics from surface features and turbid water plumes. Furthermore, total atmospheric transmittance should be taken into account and wavelengths should be selected outside the strong atmospheric absorption regions. In general, the study confirmed some conclusions from several earlier publications but also found differences. Several absorption features are found in the SWIR region, as in the literature, but the exact position of the features can be slightly different. For instance, Garaba and Dierssen^11^, mentioned features centered at 931, 1215, 1417 and 1732 nm whereas we have found them centered often at 1192, 1413, 1730 nm. The small shifts in the wavelength should be examined further. Possible explanations could be the specific type of plastic with coatings and additives or the lab conditions.

Taking into account atmospheric transmittance, absorption features around 1192–1215, 1660 nm and 1730 nm seem most suitable for polymer discrimination. The feature around 1730 nm can serve also for separating plastics from wood. Wood with its brownish color is difficult to discriminate from the water and the brownish plastics in the VIS, but could be distinguished from plastics in the SWIR based on the absorption features at 1729 nm where wood does not have any feature. Marine harvested samples from the port of Antwerp show that the features can be less pronounced in real weathered conditions. Spectral information in the SWIR region is valuable for solid plastics such as the orange tube, but very thin and transparent plastic bags will be hard to identify. The degree of weathering and bio-fouling and their impact on the spectral reflectance should be investigated further. Although no characteristic plastic absorption features are present in the VNIR, this spectral region should also be considered and might aid in the identification of marine plastic litter. In the visible part of the spectrum, colored plastics can be confused with the water itself. Brownish plastics will be difficult to discriminate from a turbid plume, blue plastics will be more difficult to discriminate in clear oceanic waters. The visible spectral region might still be useful to discriminate plastics with a different color than the water and to discriminate colored plastics from white caps, even when submerged; for instance, in case of an orange placemat it can be differentiated till a submersion of 16 cm.

In the NIR, from 850 to 900 nm we did not observe a very prominent effect of the color and plastic reflectance is generally higher than clear and turbid water reflectance, because pure water absorption is increasing. Moreover, slightly submerged plastics can be detected at this range as well, in the case of an orange placemat, 5 to 10 cm depending on the TSM concentration. 1070 nm is the best wavelength to discriminate plastics from extremely turbid water and also detecting submerged plastics less than 5 cm. Still, white caps, when present, complicate the retrieval of plastics in this spectral range. Floating macro algae and aquatic vegetation were not considered in this study but both might influence the reflectance at 1070 nm. Saturated macro algae have shown to have a reflectance peak around 1070 nm^37^, similar than turbid water. However, floating plastics will probably exhibit a higher absolute reflectance in this wavelength region. Aquatic vegetation might become more complicated to discriminate from the plastics in this region because of its high NIR reflectance plateau.

Spectral information in the SWIR region is valuable for solid plastics such as the orange tube, but plastic bags will be hard to identify. Several spectral regions can be exploited: maximum reflectance and specific absorption features related to the polymer type^19–22^. Considering atmospheric transmittance factor, 1280–1300 nm and 1550–1600 nm are the wavelength ranges allowing maximum reflectance through the atmosphere.

Absorption features are another criteria for developing new sensor for plastic detection, 1660 nm is suitable for polymer discrimination while 1730 nm is great for separating plastics from wood and polymers. For instance, wood with its brownish color is difficult to discriminate from the water and the brownish plastics in the VIS, but will be easy to distinguish from bags and bottles in the SWIR. Discriminating wood from solid plastics in the SWIR might be possible based on the absorption features at 1729 nm where wood does not have any feature.

Indeed, VIS, 1070, 1192–1215, 1660 and 1730 nm are most usable wavelengths for marine plastic detection; For the design of a new sensor, wavelengths can be selected on the position of the absorption feature and outside the absorption feature, at the maximum reflectance. The proposed wavelengths are also challenging from a technological point of view. The 1070 and 1730 nm wavelengths are at the limits or outside the spectral responsivity of standard indium gallium arsenide photodiodes (InGaAs) and silicon based detectors. A combination of different detectors or extending current detectors spectral responsivity will be needed.

The proposed wavelengths might not solve some issues identified in this study. It has been shown that whitecaps can complicate the spectral identification of several types of plastics. Mainly white, transparent plastics such as plastic bags will be easily mistaken for a whitecap. In this case, additional information on the shape and size of the plastics is needed.

Considering recent advances using Sentinel-2 data which were evaluated in the laboratory (see “Results” section), we believe conclusions from satellite data in previous studies are not easily transferable to other locations because of the variety in type and color of plastics, the differences resulting from immersion, wetness and from the different properties of the water itself.

All in all, object detection in visible bands and spectral detection in SWIR bands at the same time will be a comprehensive approach to detect macro-plastics in marine environment. A combination of RGB and SWIR spectral data can be assimilated into customized object based detection as well as AI algorithms which showed promising results in the detection, classification and quantification of floating and washed ashore marine litter^38–41^. Descriptors related to shape, color, size and form of plastic litter can be derived from RGB data whilst SWIR provides further information related to polymer type.

## Methods

### Laboratory and tank set-up

Spectral reflectance of the plastic targets was measured with an Analytical Spectral Devices (ASD) FieldSpec 4^42^ from the ultraviolet (UV, 350 nm) to shortwave infrared (SWIR, 2500 nm). The VNIR spectrometer has a spectral resolution of approximately 3 nm at around 700 nm. The spectral resolution in the SWIR varies between 10 and 12 nm. The reflectance R is defined as L/Ed with L the upwelling radiance and Ed the downwelling irradiance. A Labsphere Spectralon 99% diffuse was used for white referencing to derive relative reflectance measurements from the ASD. A foreoptic with an 8$^{\circ }$ field-of-view was attached to the optical fibre detector end of the ASD. Each measurement consisted of 30 scans and 5 replicate measurements were taken for each target.

**Figure 8:**
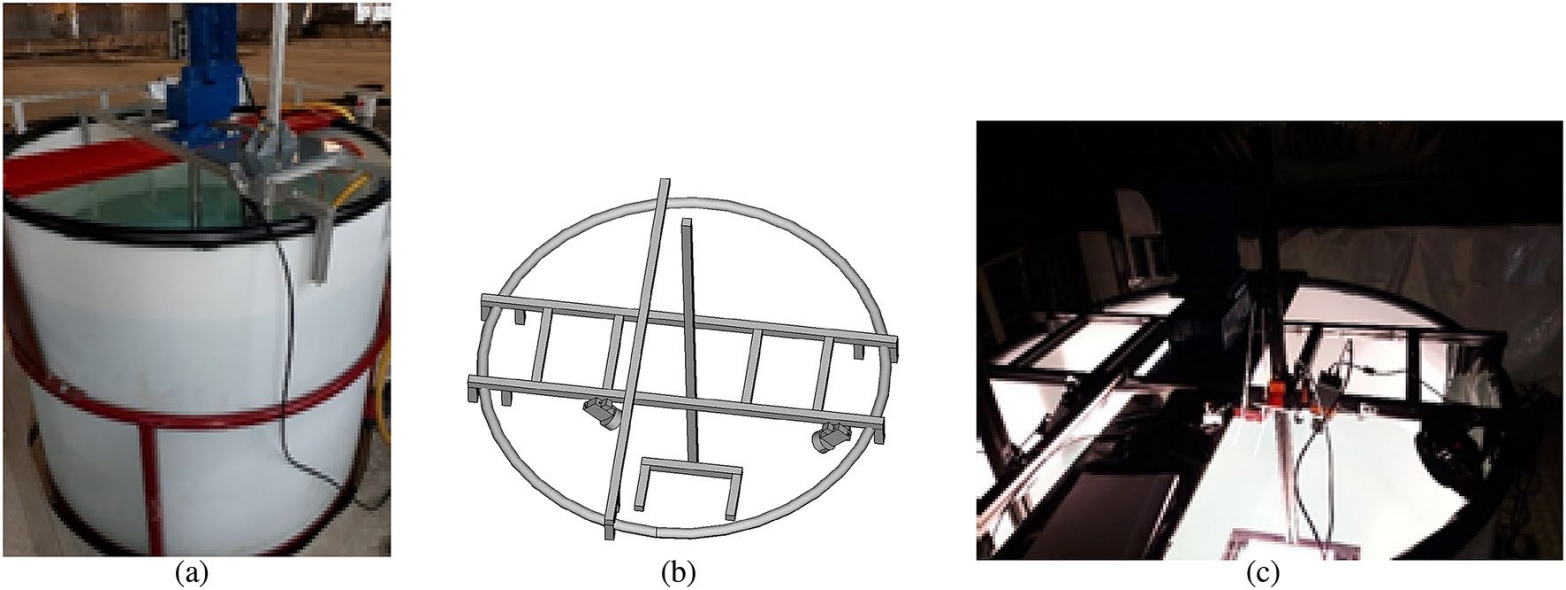
Experimental setup. (**a**) Water tank with the electrical mixer attached to an aluminium frame, (**b**) schematic of a custom-made aluminium frame with an adjustable arm for varying depth of sample below water surface with two light source attached above the water surface. (**c**) Set-up of the tank covered with black cloth.

Spectral reflectance measurements were performed in an optical calibration laboratory at VITO, Belgium and water tank at Flanders Hydraulics facility in Antwerp. The water tank of diameter 2 m and depth 3 m had a propeller attached allowing near homogeneous distribution of sediments (Fig. 8). A set of two halogen lamps (12V 50W GY9.5, Original Gilway L9389) were used to provide artificial lighting simulating sunlight. A tailor-made aluminum frame was attached to the water tank for the attachment of the spectroradiometer detector, lights and samples (Fig. 8b). The frame was also painted black so that it would not contribute to the bulk spectral signal expected from only the plastic targets. It also consisted of an adjustable arm with a holder for the targets marked had predetermined depth markings. A black cloth was used to create a dark surrounding over the water tank to mitigate stray light from the laboratory surfaces.

An additional splice correction^42^ was applied to the data. This correction has been applied to remove jumps in the spectra due to overlaps by different detectors at 1000 and 1800 nm. The difference between 1000 and 1001 nm was used to correct the VNIR data (350–1000 nm), whilst the difference at 1800 and 1801 nm was used to correct the SWIR-2 spectra (1800–2500 nm)^34^.

In this study we wanted to simulate different conditions of water turbidity by taking spectral measurements of the plastics in waters with low/none, moderate and high TSM concentration. To achieve these concentrations of moderate and high TSM, we used clay sediments gathered from a tidal Deurganckdok in the Belgian harbor of Antwerp. These clay sediments had a median particle size D50 of $11\pm 0.3\%$ $\upmu$m which ranged between D10 of 2 $\upmu$m and D90 of 51 $\upmu$m. After measurements at low concentration ($5.3\pm 1.7\%$ mg/l), a small amount was added to the water tank to obtain a moderate amount ($75\pm 4\%$ mg/l) and then a final addition was done to reach the high level ($321\pm 6\%$ mg/l)^43^.

### Plastic specimen

Optical properties were measured on a set of harvested/weathered and virgin plastics. We gathered weathered litter from the port of Antwerp consisting of plastic bottles, plastics bags, plastic pellets, rope and wood. The virgin plastics included a black plastic waste bag, plastic bottles (PET), polyester ropes, placemats and ropes (PP, PE) in different colors. Knaeps et al.^43^ explains the data set^44^ in more details like including picture of each sample, selection of samples for the tank experiment based on statistical analysis for all 47 hyperspectral reflectance measurements. The plastic specimen investigated were described^43^ using the widely accepted recommendations (GESAMP, 2019)^45^. Polymer type identified by looking at the label imprinted at the side or bottom of the object. Additional descriptor like the apparent colour, shape, age and form of the plastics were determined by visual inspection. We classified the age based on the samples having been collected in the natural environment (weathered) or recently purchased for the experiments (virgin).

### Indexes driven from Sentinel-2

Recently, Biermann et al.^36^ highlighted the use of Sentinel-2 for marine plastic detection through the Normalized Difference Vegetation Index (NDVI) and Floating Debris Index (FDI). Through applying of these indexes, floating plastic aggregations in 4 different case studies at subpixel level were detected with 86 percent accuracy. They monitored an NDVI range from 0 to 0.2 and an FDI range from 0.02 to 0.06 for plastics and used it as main criteria for classification. Here we show the use of the marine plastics database to test the algorithm. To reach this aim, first we resampled our data to Sentinel-2 to make 12 Multi Spectral Instrument (MSI) bands through spectral responses of Sentinel-2 MSI^46^ and then we calculated NDVI and FDI as represented in Biermann et al.^36^.

### Whitecap spectrum and turbid water spectrum

Breaking waves generate turbulence and entrain air at the surface, forming clouds of bubbles beneath and foamy patches on the sea surface called whitecaps^47^. Whitecaps may alter the spectral reflectance in a similar way as marine plastic litter does. They may act as false positives when trying to detect marine plastic litter from satellites, drones or fixed camera. Hence, when selecting appropriate wavelength and designing algorithms for marine plastic litter detection, it is very important to evaluate the spectral reflectance of whitecaps. Therefore, whitecap spectra have to be considered next to the plastic responses. A third order polynomial has been used to generate whitecap spectrum^27^:

1 $$\begin{aligned} R_f= 0.47 x^3 - 1.62 x^2 + 8.66x + 31.81, \end{aligned}$$

where $x= log (\alpha _w)$ and $\alpha _w (m^{-1})$ is absorption coefficient of water. The effect of water turbidity on the spectral reflectance is also taken into account because turbidity plumes might be misinterpreted for marine plastic litter patches. Algae and aquatic vegetation were not evaluated in this study because the focus of the research was on sediment dominated waters, however, both may have an impact on the retrieval. The SeaSWIR dataset^35^, consisting of 97 water reflectance and TSM measurements at three estuarine sites (Gironde, La Plata, Scheldt) was used as water dataset. Moreover, pure water absorption coefficient^30,33^ and total one way atmospheric transmittance simulated using Modtran radiative transfer code^48^ for a platform height of 800 km, a nadir view, a visibility of 17 km, a water vapour content of 2.5 $\text{{m}}^{-1}$ and a rural aerosol.

## Data Availability

For reproducibility and also using measurements as a reference, all data in this research are published in an open access format in 4TU. ResearchData^44^.
